# Further Evidence of Mutational Heterogeneity of the *XPC* Gene in Tunisian Families: A Spectrum of Private and Ethnic Specific Mutations

**DOI:** 10.1155/2013/316286

**Published:** 2013-07-25

**Authors:** Mariem Ben Rekaya, Manel Jerbi, Olfa Messaoud, Ahlem Sabrine Ben Brick, Mohamed Zghal, Chiraz Mbarek, Ashraf Chadli-Debbiche, Meriem Jones, Mourad Mokni, Hamouda Boussen, Mohamed Samir Boubaker, Becima Fazaa, Houda Yacoub-Youssef, Sonia Abdelhak

**Affiliations:** ^1^Laboratoire de Génomique Biomédicale et Oncogénétique (LR 11 IPT 05), Institut Pasteur de Tunis and Université de Tunis El Manar, El Manar I, 2092 Tunis, Tunisia; ^2^Département de Dermatologie, Hôpital Charles Nicolle de Tunis, 1006 Tunis, Tunisia; ^3^Service d'ORL et de Chirurgie Cervico-Faciale, Hôpital Habib Thameur, 1008 Tunis, Tunisia; ^4^Unité de recherche “Troubles Héréditaires de la Kératinisation” UR 24/04, Hôpital La Rabta de Tunis, 1007 Tunis, Tunisia; ^5^Département de Dermatologie, Hôpital La Rabta de Tunis, 1007 Tunis, Tunisia; ^6^Département d'Oncologie Médicale, Hôpital Abderrahman Mami, 2080 Ariana, Tunisia; ^7^Laboratoire d'Anatomie Pathologique Humaine et Expérimentale, Institut Pasteur de Tunis, 1002 Tunis, Tunisia

## Abstract

*Xeroderma Pigmentosum* (XP) is a rare recessive autosomal cancer prone disease, characterized by UV hypersensitivity and early appearance of cutaneous and ocular malignancies. We investigated four unrelated patients suspected to be XP-C. To confirm linkage to *XPC* gene, genotyping and direct sequencing of *XPC* gene were performed. Pathogenic effect of novel mutations was confirmed by reverse Transciptase PCR. Mutation screening revealed the presence of two novel mutations g.18246G>A and g.18810G>T in the *XPC* gene (NG_011763.1). The first is present in one patient XP50NEF, but the second is present in three unrelated patients (XP16KEB, XP28SFA, and XP45GB). These 3 patients are from three different cities of Southern Tunisia and bear the same haplotype, suggesting a founder effect. Reverse Transciptase PCR revealed the absence of the *XPC* mRNA. In Tunisia, as observed in an other severe genodermatosis, the mutational spectrum of XP-C group seems to be homogeneous with some clusters of heterogeneity that should be taken into account to improve molecular diagnosis of this disease.

## 1. Introduction 

*Xeroderma Pigmentosum *(XP) is a rare monogenic autosomal recessive DNA repair disorder. It is characterized by an extreme sun sensitivity especially to Ultra Violet Radiation (UVR) that induces cutaneous and mucous membranes cancers of the eyes and mouth [1].

XP is the archetype of an expanding family of Nucleotide Excision Repair (NER) diseases where XP cells were found to have defects in seven of the NER pathway proteins (XPA to XPG) [2].

In addition, an XP variant group (XP-V) involving a DNA translesional synthesis (TLS) and polymerase Pol eta (*POLH*) has also been described [3]. XP-V patients have a normal NER level, but their cells are hypermutable following UVR.

This disease is rare in the United States and Europe (1/100000) [4] but relatively more frequent in Japan (1/22000) [5] and in North Africa [6] especially in Tunisia with 1/10000. This high frequency might be due to the high rate of consanguinity in Tunisia (29.8%) especially among families with autosomal recessive diseases (78.4%) [7] and to founder effects reported in *XPC* [8], *XPA* [9], and *XPV* genes (unpublished data) in the Tunisian population.

The *XPC* gene plays an important role in the early step of Global Genome Repair pathway (GGR). XPC protein is involved in the recognition of the damaged DNA via a complex composed by HR23B-XPC-CEN during GGR pathway [10]. Currently, 46 mutations have been described in this gene. The most common are deletions (36.9%), substitutions (34.7%), splicing (19.5%), and insertions (8.6%). Splice site mutations have been described, in introns 2, 5, 1, 8, 9, and 11 [11].

Molecular investigations of the XP-C group showed that it is the major group among the five North African countries called the Maghreb [6, 8, 12, 13]. Indeed, among the studied patients, about 87% of them shared the recurrent founder mutation c.1643_1644delTG (p.V548AlafsX25) [6]. This was confirmed by its high carriers frequency estimated to be 1/250 in Morocco [12]. Having a homogeneous mutational spectrum for the *XPC* gene has greatly simplified the molecular diagnosis of XP-C in the region [6, 8, 13]. Furthermore, two other private mutations have been recently reported in North African patients [6]. In this study, we have further enlarged the mutational spectrum of the *XPC* gene with the identification of two novel gene alterations.

## 2. Patients and Methods

### 2.1. Patients

This study was performed on 4 consanguineous Tunisian families originating from Southern Tunisia and encompassing 15 individuals. Among them, 4 patients (2 males and 2 females) were suspected to be XP-C. The age of the investigated XP patients is heterogeneous (ranging from 7 to 22 years). Consanguinity was confirmed in all families.

### 2.2. Methods

Informed consent was obtained from all available family members or the parents of minor children. Families were interviewed using structured questionnaire to collect information about family history, consanguinity, affected members, and associated diseases. This protocol was approved by the Ethics Committee of the Pasteur Institute of Tunis.

#### 2.2.1. DNA Extraction

DNA was isolated from peripheral blood leukocyte using salting out method [14] or Qiagen kit DNA extraction. Genotyping for 14 available individuals was determined using 3 microsatellite markers spanning a 1.43 Mb interval that overlaps the *XPC* gene region (D3S3602, D3S1585, and D3S3613). Microsatellite markers were selected from the genetic maps available on NCBI browsers and the CEPH genotype database (http://www.cephb.fr/en/cephdb/) on the basis of their heterozygosity percentage and closeness to the *XPC* gene. Genomic DNA amplification was carried using specific primers (Table 2), and the fragments were amplified in a volume of 25 *μ*L containing 10 ng of DNA, 10X PCR buffer, 0.2 mM of each dNTP, 1.5 mL of MgCl_2_, 0.5 *μ*M of one of each primer, and 0.5 U Amplitaq DNA polymerase (Invitrogen). Fluorescent PCR products were run on the Genetic Analyzer and analyzed using the Gene Mapper software.

#### 2.2.2. Mutation Analysis

Exons 4 to 16 and their flanking regions were amplified from genomic DNA. Primer pairs were generated using Primer 3 http://frodo.wi.mit.edu/ and UCSC *In Silico*-PCR (http://genome.ucsc.edu/cgi-bin/hgPcr) (Table 3). PCR conditions used in our laboratory were previously described [8]. PCR products were directly sequenced using the ABI 3130 Genetic Analyzer. Nomenclature mutations were performed using the NG_011763.1 according to the HGVS version 2.0 (Mutalyzer 2.0.beta-26).

#### 2.2.3. RNA Extraction and RT-PCR

In order to assess the impact of the identified mutations at the RNA level, total RNA was isolated from leukocytes using trizol-chloroform purification and isopropanol precipitation [15]. The only biological material that was available for RNA extraction for the XP45GA and XP50NEF patients was 1 mL of whole blood frozen in 10% Dimethyl Sulfoxide (DMSO) at −80°C, 4 years ago. RT-PCR of the control was performed on a fresh blood sample for a healthy volunteer. 

The RNA (0.4 *μ*g) was reverse transcribed in a final volume of 20 *μ*L using the reverse transcriptase (Ref 1785826, Roche), OligodT (Ref 27-7858-01, Pharmacia), Buffer 1X (Ref 1785826, Roche), dNTP 1 mM (Invitrogen), and the Rnase inhibitor 40 U/*μ*L (1785826, Roche). The cDNA corresponding to the *XPC* gene was amplified using a new couple of primers (Table 2). In order to evaluate the quality of the cDNA amplification, a PCR for a housekeeping gene *GAPDH* was conducted using primers showed in Table 2 (Figure 3).

## 3. Results

### 3.1. Clinical Findings

All investigated patients had the classical skin abnormalities of XP including xerosis, skin atrophy, and abnormal pigmentation with no neurological abnormalities. In our study, XP symptoms began at a mean age of 39 months. Skin hyperphotosensitivity from UVR was present in all patients. The specific poikilodermic pigmentation pattern (association between achromic spots and poorly limited pigmented maculae) covered the majority of the sun-exposed areas. The mean age at onset of the first skin cancer was 12 years (range 8 to 17). Basal Cell Carcinoma (BCC) occurred in 3 patients (XP28SFA, XP45GB, and XP50NEF). Among them two patients (XP50NEF and XP45GB) developed melanoma (MM) and Spindle Cell Carcinoma (SCC) (Table 1).

In addition, patients XP45GB and XP28SFA developed, respectively, a thyroid cancer and a benign articular tumor of the knee (Table 1). For the patient XP45GB, clinical and ultrasound exams showed bilateral multinodular thyroid without hormonal dysfunction (normal rates of T4, T3, and TSH). After thyroidectomy, histological analysis confirmed thyroid carcinoma. No metastasis was found.

### 3.2. Genetic and Molecular Results

**Figure 1:**
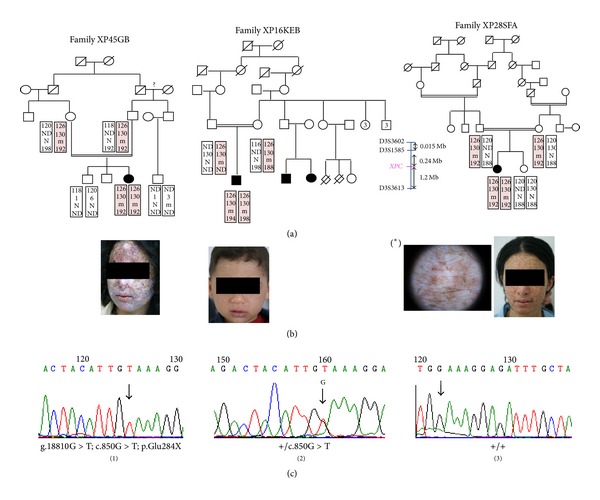
(a) Pedigrees and haplotype analysis for the XP45GB, XP16KEB, and XP28SFA patients. The disease haplotype is indicated by shading. m, mutant allele; N, normal allele; ND, not determined. (b) Clinical photographs of investigated XP-C patients showing clinical variability despite genetic homogeneity. (*) Dermoscopic examination of XP28SFA patient showing polymorphic pigmented and achromic macula. (c) Genomic DNA sequence showing the G to T substitution in the exon 7 of the *XPC* gene in homozygous state (1) or in a heterozygous parent (2) which is compared to the wild-type sequence of a control (3).

**Figure 2:**
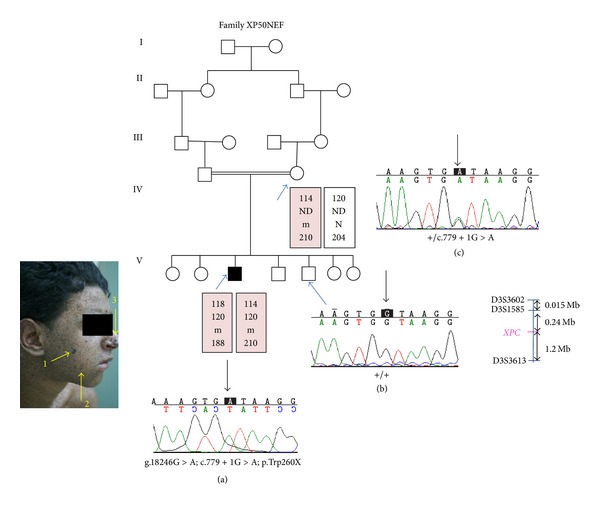
(a) Pedigree and haplotype analysis for the XP50NEF family. The disease haplotype is indicated by shading. m, mutant allele; N, normal allele; ND, not determined. (b) Clinical photograph of XP50NEF patient (1: melanoma; 2: angiogranuloma; 3: BCC). The sequence electropherogram of the sense strand in exon 6 of *XPC* gene showing the G>A substitution in homozygous state (a) in comparison with the wild type sequence (b) or in a heterozygous parent (c).

For molecular diagnosis, we first screened the recurrent founder mutation for all XP-C suspected patients. Since this gene alteration has not been identified, linkage to the *XPC* gene was performed. Fourteen individuals were genotyped with 3 informative microsatellite markers. All XP-C patients showed homozygous genotypes for the D3S1585 marker. Three patients (XP16KEB, XP45GB, and XP28SFA) shared the same founder haplotype (126–130), respectively, for D3S3602 and D3S1585 markers (Figure 1), whereas patient XP50NEF presented a private haplotype (Figure 2). Therefore, sequencing of exons 4 to 16 was performed for all XP patients. Two novel variations were identified. The first one is a splicing mutation (g.18246G>A; c.779+1G>A) present at a homozygous state in patient XP50NEF. It likely abolishes the exon 6 splicing donor site as shown by the following bioinformatic tools: “BDGP” (http://www.fruitfly.org/seq_tools/splice.html) and “Human splicing finder mutation” (http://www.umd.be/HSF/). This mutation probably leads to a premature stop codon at position 260 that results in a truncated XPC protein (p.Trp260X). To note, this mutation is not present in the public dbSNP database (http://www.ncbi.nlm.nih.gov/snp). In addition, Mendelian inheritance of this mutation was confirmed (Figure 2).

The second mutation is a G to T substitution at position 14521 of the genomic DNA (g.18810G>T; c.850G>T) present at a homozygous state in (XP28SFA, XP16KEB, and XP45GB) patients. This founder mutation probably leads to a premature stop codon at position 284 of XPC protein (p284X).

**Figure 3:**
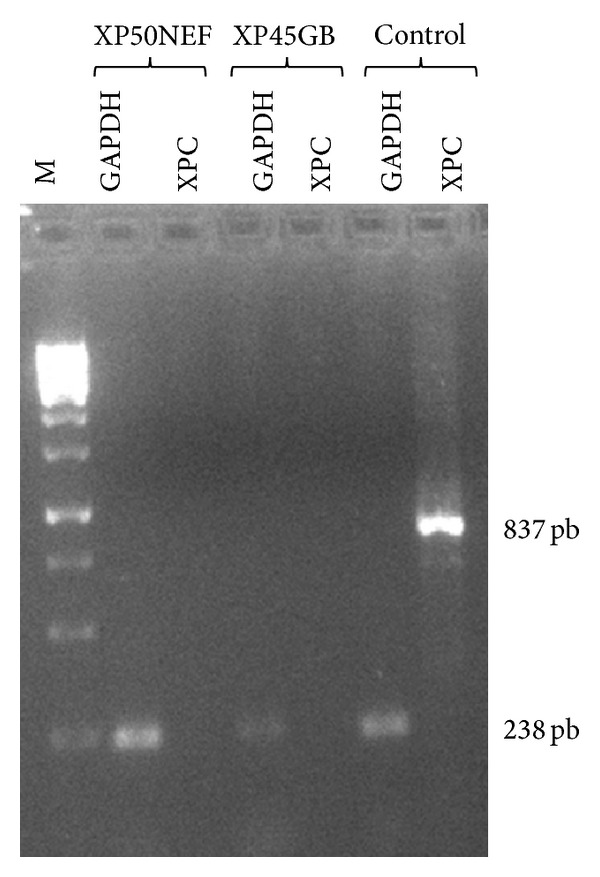
Agarose gel electrophoretic analysis of the RT-PCR showed the absence of the *XPC* cDNA amplification for XP50NEF and XP45GB patients compared to healthy control. The cDNA of the* GAPDH *gene was successfully amplified for all individuals. The variability of intensity of amplification between XP45GA patient and control is due to the quality of conservation of the blood sample. (M: 1 kb DNA ladder molecular size marker (GeneRuler)).

RT-PCR results showed the absence of *XPC* mRNA for (XP50NEF and XP45GB) patients having, respectively, the c.779+1G>A and c.850G>T mutations, although we succeeded the amplification of the* GAPDH* cDNA (Figure 3). 

## 4. Discussion

XP is a highly heterogeneous disease at clinical and genetic levels. XP-A and XP-C are the most frequent groups, especially in Southern Europe and North Africa [4, 6]. Previous studies have shown that all XP-C Tunisian patients shared the same mutation c.1643_1644delTG (p.V548AlafsX25) affecting exon 9 [8]. We report here two novel mutations in XP-C Tunisian patients. All investigated patients have classical symptoms of XP with varying severity. BCC occurred in three among four patients confirming that it is the most frequent skin cancer observed in XP patients [16]. Among the studied patients, two (XP28SFA and XP16KEB) have benefited from a complete UVR protection thanks to the help of the patients support group “Helping Xeroderma Pigmentosum children” (http://www.xp-tunisie.org.tn/) (Table 1). Consequently, XP28SFA patient developed one BCC at the age of 17 years that is higher than the median age at onset of Non-Melanoma Skin Cancer (NMSC) in XP patients (9 years) [17]. Regarding XP16KEB patient, until the age of 7 years, he did not develop skin cancer. Melanoma occurred in two patients XP50NEF and XP45GB at the age of 17 years which is lower than the median age at onset of melanoma (22 years) in XP patients [17]. Regarding the XP45 GB patient, she was treated by retinoid at age 13 during a period of 3 years; after that the number of NMSC was increased. At age 16, she developed nodular thyroid. During the last consultation at age 17, we discovered that the patient showed resistance to cisplatin chemotherapy used to treat multiple spindle-cell carcinomas of the face. The precise role of retinoid in cancer inhibition has not been clearly demonstrated in XP patients. Recent studies have shown a key role of GGR pathway especially XPC protein to repair cisplatin and eventually UV lesions in melanomagenesis [18, 19]; further investigation possibly will explain the resistance of XP-C patient to chemotherapy.

In addition to skin cancers, XP45GB developed a thyroid cancer. She was not treated by X-rays or radiotherapy. According to the genetic questionnaire filled by the interview of the parents, there is no other cancer case in the family. Generally, XP patients can rarely develop internal cancer because they die at an early age. A recent study demonstrated that multinodular thyroid was the most frequent kind of internal tumor in XP-C patients [16], while adenocarcinoma of the thyroid was reported in only one 18-year-old XP-C patient [20]. This might be due to the accumulation of oxidative damage in the thyroid [21].

In the absence of the recurrent mutation *XPC* c.1643_1644delTG, genotyping and haplotype analysis showed that all XP-C patients are homozygous for the closest marker to *XPC* gene, D3S1585. Sequencing and mutation analysis confirmed the presence of the two novel mutations. Thus showing that the D3S1585 marker is the most informative marker.

In order to test the impact of the two identified mutations at the functional level, we performed an RT-PCR experiment for patients when RNA was available. For XP50NEF and XP45 GB patients, *XPC* mRNA was absent. The same result was obtained in XP-C patients bearing the recurrent mutation (c.1643_1644delTG) in exon 9. The presence of these mutations probably reduces transcription of the *XPC* gene or alters the stability of its mRNA which could be degraded by the NMD system (Nonsense Mediated mRNA Decay) [22]. 

Although the two patients XP45GA (having a nonsense mutation) and XP50NEF (having a splice site mutation) underwent a low UV protection and have the same age, the second patient XP50NEF had a milder phenotype than the first (Figures 1 and 2). A small amount of normally spliced mRNA of the *XPA* gene is sufficient to explain relatively mild clinical features of XP-A patients [23]. Previous study reported that XP-C patient with a splice site mutation has a very low level of normal *XPC* mRNA transcript (3%) resulting in 29% of normal level of normal sized XPC protein [24]. For the XP50NEF patient, cell lines are not available to confirm the presence of a small amount of *XPC* mRNA isoforms.

Genetic counseling was performed for four related individuals. The sister of XP28SFA and the brother of XP45 GB were healthy with a homozygous genotype, whereas the cousin of XP45 GB and the brother of XP50NEF were healthy heterozygous carriers (Figure 1).

A recent study showed that in Tunisian patients among 174 genetic diseases with identified molecular defect, 73 (41,9%) were founder mutations. For the majority of them, geographic distribution is limited to a region of the country [25]. A similar situation is observed in our study where the c.850G>T founder mutation seems to be restricted to the South of Tunisia. Knowing the disease features in a given population is important to offer a suitable management of the patients and their families [25]. As XP-C is the most common group in North Africa with a high morbidity and an early mortality, primary diagnosis is important. As XP-C patients do not show neurological impairment, they could live a nearly normal life if they benefit from an early full protection against sunexposure. This is the case of patient XP28SFA who has been protected during early infancy and is now following “normal” university studies. But other clinical exams should be programed especially thyroid ultrasound for early detection of internal cancer. Since dermatological exam could not confirm exactly the XP group, specifically for young patients, molecular diagnosis is necessary to confirm clinical diagnosis and to propose genetic counseling. 

The high frequency of the same founder mutations in patients from North Africa has greatly simplified the molecular diagnosis of XP syndrome in Maghreb region. Considering the efforts and costs required for the unscheduled DNA synthesis on cultured fibroblasts, we therefore suggest, for suspected XP-C patients without *XPC*-p.Val548AlafsX25 mutation, genotyping analysis to confirm linkage to *XPC *gene especially for consanguineous or endogamous families. If linkage to *XPC* gene is confirmed, the second founder mutation, c.850G>T in exon 7, should be investigated. In the absence of this mutation, sequencing of exon 6 must be performed. This exon contains three different mutations (c.652delT, c.658C>T, and c.779+1G>A) described in North African patients. Cascade screening should be proposed to all the families in which an index case has an identified mutation [26, 27].

## 5. Conclusion 

Our results show that private mutations, ethnic specific mutations, and recurrent founder mutations could explain the mutational heterogeneity of XP-C group in Tunisia. In addition, our findings provide a valuable tool for molecular diagnosis and carrier screening of this severe genodermatosis at the regional level where endogamy is still culturally favored.

## Figures and Tables

**Table 1:** Microsatellite markers surrounding the *XPC* locus.

Microsatellites markers	Physical distance (pb)	Genetic location (cM)	Heterozygosity	Number of alleles	Primers sequence	Allele size range	Fluorescence
D3S3602	13.926.066–13.926.191	31.40	57.69%	7	F: AAAATCCTAACCCAAAATGT R: ATCAGAAAATAACAGAGGGC	114–132	FAM

D3S1585	13.941.728–13.941.855	33.00	57.14%	8	F: TGCACGAGCCAGAAGT R: TTGGACTGCTGAGGGG	126–144	NED

D3S3613	15.361.998–15.362.181	35.70	78.57%	8	F: CATCTATGTGGCAATCGG R: CAGCATTTGTTGTAGGGACT	172–208	FAM

**Table 2:** Sequence primers used for PCR amplification of *XPC* and *GAPDH* genes.

	PCR	Primer Sense	Primer Anti-sense	*T*_*m*_ °C
XPC	Exon 4	ATGCCTCACTTCCTCCTTCC	CACTTTGATACTCAGTCCTGGTCCC	55
	Exon 5	GATTCACTGTCATCCGAGGAGAAG	CAAAGGCTCAGAGAGAGTAAGAAACTTG	55
	Exon 6	TGAAAGACAAGACCAAAACAAAAACAG	GACCTGAACCCAGCCTCTGAG	55
	Exon 7	CTCCCTCTTTTTATTTTCTTGGCTG	GGTGCCTGTAGGCATTTGATAAAGC	55
	Exon 8	TTGAACAAGCACCATAACAAACAAC	TGCCCAAGTCTTCCCTAACACAG	55
	Exon 9	CCAGGGTGTCTTATAAAGAGG	CAAGGCCTTACCTCCAAG	55
	Exon 10	CCTTGGCTCCACCATCTGTTG	CCCTGTAACTGTTTTTCCCCTGC	60
	Exon 11	AGATTAGGGTTTGTAAGTGGACACATC	GGACTGGGAGGCTCATCATCAC	55
	Exon 12	CTGGTAGGTGTGTTCTGAGGGTTC	CGGTGTAGATTGGGCAGGTTC	60
	Exon 13	GGCAGCATCAGAAGGGCTCAG	AAATCCAGTGTAACATCCTGAAAATTG	60
	Exon 14	AGGCTGGATAGGGGCTTTCAC	CCTGCTGTATTCAGTGCTCGCTC	60
	Exon 15	CCACTAAAGATTTTGGAGTCAGTAACG	ACAGGGCTTGGGGCAGAAGAG	55
	Exon 16	CCCTTGTCCTCCCAGAGTTACAC	ATGCTGCCTCAGTTTGCCTTC	60
	cDNA	TTGAAGAACTTAGTGAGCCTGTG	GCTGGGTTGCCTTCTCCT	60

GAPDH	cDNA	GAGTCAACGGATTTGGTCGT	TTGATT TTGGAGGGATCTCG	60

**Table 3:** Clinical features of the XP-C patients.

Patients code	Sex	Age at first consultation (years)	Age (years)	City geographic origin	Age at onset of the first skin tumor (years) (number)			Internal cancer (age at onset)	Protection from UVR
					MM	BCC	SCC		
XP16KEB	M	2	7	Kebelli	—	—	—	—	+++
XP28SFA	F	3	21	Sfax	—	17 (1)	—	Benign articular tumor of the knee (18)	+++
XP45GB	F	2	18	Gafsa	17 (1)	8 (>20)	9 (>10)	Thyroid cancer (16)	+
XP50NEF	M	6	22	Nefta	17 (1)	11 (>5)	17 (1)	—	+

MM: melanoma; BCC: Basal Cell Carcinoma; SCC: Spino Cell Carcinoma; +++: high protection; +: low protection.
